# Foreign Hospitals

**Published:** 1896-09-19

**Authors:** 


### FOREIGN HOSPITALS.

### HELSINGFORS, FINLAND.—THE SURGICAL HOSPITAL.

In the old General Hospital at Helsingfors only a few wards were allotted to surgical cases; and this accommodation eventually proved to be altogether inadequate to meet the growing demands for space and up-to-date sanitary arrangements. The present hospital was therefore built and opened by Government in November, 1888. In its construction the State grudged neither money nor land, the site being an ideal one for such an institution. It stands in its own grounds, at the top of a high hill, or rather rock of solid granite, within a short distance of the sea, of which a lovely view is had from the south pavilion. the numberless small islands dotted along the coast, with the lighthouse in the distance, forming an enchanting panorama. Built on the pavilion system, the buildings consist of (1) main block, (2) administrative block, comprising kitchen and housekeeping department and various offices, (3) isolation block, and (4) engine-house. The main block is composed of a central division with a couple of wings, a north and south pavilion, and in a well-lighted and ventilated basement are situated the heating, lighting and ventilating apparatus, a laboratory, and the porters' rooms. On the first floor, to the left of the magnificently proportioned entrance-hall, there is an office and house-surgeon's rooms. The matron's charming quarters lead from a separate vestibule, and consist of sitting-room, bedroom, and small kitchen. To the right of the hall lies a large room for students, and also the out-patient department; the latter consists of waiting, reception, and a small operating-room; the waiting-room is sub-divided at the upper end near the large windows to form a distinct department for dentistry and diseases of the ear, under a special assistant surgeon, and adjoining this there is the dispensary and a small laboratory. From the hall two broad stone staircases lead to the second floor, where to one side lie the house-surgeon's and dressers' rooms. The house-surgeon holds the position of the assistant surgeon in England, but he lives in the hospital; the post is a permanent one. On this floor the senior surgeon's waiting and consulting rooms are placed, the latter being also the library, and, on occasions, serving as board-room. A registrar's office leads into a room where instruments, splints, and other surgical appliances not in daily use are kept, and finally the theatre is reached. This room, 5 metres (1 met. = 3 ft. 3½ in.) in height, is fitted up on the latest principles, the surgical appliances being of the newest. It is lighted by four large windows, forming a wide bow at one end, besides a top light. Both gas and electric light are used on dark winter days, and for the evening work. The walls are painted from floor to ceiling, the floor being painted asphalte. Against two walls stand a white china washing apparatus, with hot and cold water laid on; along another rows of benches for students are placed, this differing from the amphitheatre in English hospitals; the three operation tables, together with the instrument tables, cases, and sprays being of enamelled iron alone, or of enamelled iron with glass. They are easily cleaned, and, if necessary, can be placed without risk of damage in the disinfecting chamber. The operation tables are constructed with hinges, and the head can be easily raised or lowered. The tops of the instrument tables consist of trays of plate-glass, in which the instruments lie in disinfectants, another piece of plate glass forming a cover. These tables, as well as the sprays, are on indiarubber castors, and thus are easily rolled about. The cleansing and disinfecting of the instruments is the duty of a staff nurse, who is alone held responsible for this work, one or two probationers being generally told off as her assistants. Close to the theatre the lift is worked by hydraulic pressure, and here it may be well to mention the admirable arrangement for the removal of patients to and from the wards. Being placed on beds on indiarubber castors, they are rolled by porter or nurse into the lift, thence into the waiting-room, to await their turn on the operation table, the return journey being performed in the same manner. The transit from ward to theatre is thus made easy for patient and nurse. The waiting-room is separated by a double wall from the theatre to prevent any sounds from penetrating. Opposite the waiting-room is a room for storing and preparing bandages and dressings, a large slab of polished granite in the centre serving as a table.

The hospital has 156 beds, arranged in thirteen divisions, each under a staff nurse, the thirteenth division being the isolation block. The wings of the main block, both lower and upper floor, are on the same plan, one wing containing male, the other female patients. Each floor forms a division, the four collectively called the central division to distinguish them from the pavilions. The division is composed of four wards, varying in size, and containing one, two, or three beds. These are for paying patients of two classes, the first-class rooms being excellently furnished, and looking very homelike. The second Class rooms are rather simpler in arrangement. Beside the wards is placed the nurse's bed-room, opening into a vestibule or anteroom, and leading from the passage there is a ward kitchen and bath-room, and on the opposite side of the passage a lavatory and lift. On the upper floor of the south wing the biggest ward has been turned into an operation-room for abdominal sections, its appointments, on a smaller scale, being similar to those of the theatre.

The pavilions are connected with the main block by covered ways 24 metres in length, and broad in proportion, with windows along each side, and floors of painted asphalte. Each pavilion is divided into four divisions, two on each floor, and as in the wings of the central block, the men's wards are on one side and the women's on the other, one ward on the women's side being reserved for children.

A division consists of a ward of twelve beds, two special wards, nurse's bed-room, ward kitchen, day-room, bath-room, and lavatory. A small space between the special wards contains a store cupboard for ward requisites and linen on one side and a lavatory in connection with the special wards on the other. The large lobby between the divisions is used as a dining-room by patients able to get about. The ward kitchens are also shared by each division in common, one room being used as kitchen, the other as ward-maid's room.

No dressings are allowed in the wards except in cases where moving would endanger the patient. One of the day-rooms at the end of the large ward is fitted up for use by both divisions as a bandaging and dresser's room, and patients are taken on their roller bedsteads to have their wounds attended to by house surgeon or dresser. This method takes up much time, besides causing extra fatigue in many cases; but from a sanitary point of view it is good and it certainly prevents much litter in the wards and the highest antiseptic efficiency is ensured. The wards are admirably lighted and ventilated, the proportion of air to each bed being 52·3 cubic metres. The wards are 4·5 metres in height and the floor space to each patient is 11·5 square metres, the proportion of window to floor space being as 1 to 5. The walls are distempered with a high-painted dado, the floors of polished oak parquet, the ceilings varnished wood. An improvement is gradually being carried out in wards and ward offices by the walls and ceilings being enamelled. Broad wooden verandahs run the length of the south wall of each division, and on summer days patients can be rolled out in their beds to enjoy the fine air off the sea. The sides are provided with light roller blinds made of thin laths of wood which can be rolled up or down and forming an excellent protection from the wind. Sometimes in very warm weather patients from the children's wards are wheeled out in their cots and remain day and night in the fresh air, this treatment having been found most efficacious in cases of tuberculosis. The appearance of the wards is pleasant and bright, the windows are provided with white cotton curtains, and the iron bedsteads with red and white quilts. The latter stand a good distance out into the room. Between each bed, instead of an English locker, stands a lightly-made enamelled iron table with a shelf underneath, covered with a white cloth and hidden by a curtain. The men and boys look quaint in their hospital dress of blue cotton trousers and long grey woollen coats or gowns. The women wear a skirt and loose jacket, also of blue cotton material, occasionally in cold weather adding grey gowns like the men.

The hospital is heated by hot water pipes, with radiators at intervals in the passages and down the sides of the wards. The heat in each radiator can be moderated separately. Some of the rooms in the main block and the housekeeping block have the ordinary Finnish stove heated with wood.

In a country like Finland, with its rigorous winter, lasting the greater part of the year, where double windows are a necessity, artificial ventilation has to be resorted to. The fresh air is introduced through a small building, with six large wire-covered apertures. From thence it travels through a canal to the cellars of the main block, passing through a system of cleansing from all particles of dust and heating. It is then forced at the rate of 30,000 cubic m. per hour, by a ventilator worked by an engine, into the different parts of the building through canals in the walls, and it enters the wards through gratings placed at a height of 2 m. from the floor. The bad air is expelled through two separate apertures from each ward, one placed near the floor, the other close to the ceiling, and then it passes through another system of canals to eight separate shafts, through which it escapes.

The steam power required for heating and ventilating purposes is derived from boilers situated in the engine house and connected by two water pipes with the main block; from thence the steam passes through other pipes to the administrative block. There are 300 electric lamps for lighting purposes throughout the buildings and grounds, gas being also laid on in the greater part of the main block, wards included, and burning in the latter during the night.

The administrative block contains, on the first floor, kitchen and offices, nurses' dining-room, servants' rooms, and disinfecting-chamber on one side, laundry, bath-room, mortuary and post-mortem-room, dissecting-room, and rooms for clinical preparations on the other. On the second floor are housekeeper's and secretary's rooms and store-rooms.

The disinfecting-chamber is 3 metres in length, 1·5 m. high, and 1·25 m. broad, and thus large enough to admit of any piece of furniture in the hospital being placed inside.

Wooden trollies with double sides, and heated with hot water, convey the food from the kitchen to the wards. The food is served out from large copper pans to each patient, the ward crockery, such as plates and mugs, being kept in the ward kitchen, where it is arranged in neat racks along the walls.

The nurses' bed-rooms open into the large wards. They are allowed their own furniture, and, with pretty knick-knacks about on walls and tables, these rooms look most inviting, and many English nurses would envy their Finnish sisters these quarters.

The isolation block is built of wood, in one storey, with a broad verandah for patients towards the west. It contains six wards, with 14 beds altogether, and the staff nurses' bed-room, kitchen, two bath-rooms, two lavatories, and linen-room, the latter with an operation table, which is used for dressings and minor operations. The floors in the wards and passages are asphalted, the walls being of wood panelling. Each ward can be completely isolated.

The staff consists of senior surgeon, resident assistant surgeon, three house surgeons, and four dressers.

